# Supplementary material for: Preparation of Succinoglycan Hydrogel Coordinated With Fe3+ Ions for Controlled Drug Delivery
Source: Polymers (Basel). 2020 Apr 22;12(4):977. doi: 10.3390/polym12040977 (PMC7240483; doi:10.3390/polym12040977)
Supplement: Supplementary file 1 [file polymers-12-00977-s001.pdf]

polymers

MDPI

Supplementary Materials

Preparation of Succinoglycan Hydrogel Coordinated With Fe^{3+} Ions For Controlled Drug Delivery

Yiluo Hu [1,†], Daham Jeong [1,2,†], Yohan Kim [1], Seonmok Kim [1] and Seunho Jung [1,2,*]

[1] Department of Systems Biotechnology & Dept. of Bioscience and Biotechnology, Microbial Carbohydrate Resource Bank (MCRB), Center for Biotechnology Research in UBITA (CBRU), Konkuk University, Seoul 05029, Korea; lannyhu0806@hotmail.com (Y.H.); amir@konkuk.ac.kr (D.J.); shsks1@hanmail.net (Y.K.); gkdurk9999@naver.com (S.K.)

[2] Institute for Ubiquitous Information Technology and Applications (UBITA), Center for Biotechnology Research in UBITA (CBRU), Konkuk University, Seoul 05029, Korea

† These authors contributed equally to this work.

* Correspondence: shjung@konkuk.ac.kr; Tel.: +82-2-450-3520

Received: 25 March 2020; Accepted: 21 April 2020; Published: date

Table of Contents

1. GPC data for succinoglycan ……..………..………………………………………… p2

2. ^{1}H NMR spectra of the succinoglycan………..………………………..………..……p3

3. Photographs of gelation test………..……………………….……………….….……p4

4. Fe-SEM analysis………..……...………………..……………………………….……p5

5. Cytotoxicity study…………………………………………………………………..p6

1. Gel permeation chromatography (GPC) analysis

Experimental section

The molecular weights of succinoglycan was carried out by gel permeation chromatography (GPC) with a separation module (Waters e2695). The column was equipped with 300 mm×7.8 mm Ultrahydrogel column with water at a flow rate of 1 mL/min. Polyethylene glycol with specific molecular weights (106, 202, 430, 1030, 4290, 5800, 12600, 26300, 44000, 222000, 450000 Da) were used as calibration standards.

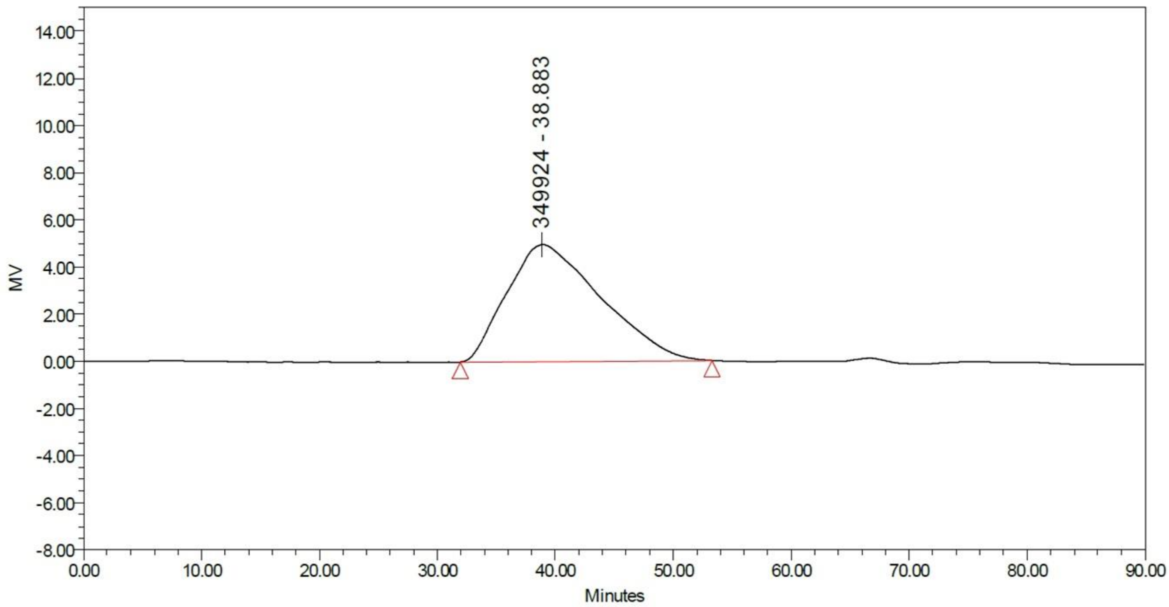

Figure S1. Determination of the molecular weight of succinoglycan via GPC.

Table S1. The molecular weight and GPC measurement of succinoglycan.

Sample Name	Mn	Mw	MP	Polydispersity	% Area
Succinoglycan	108571	354839	349924	3.268	100

2. ^{1}H NMR spectra of the succinoglycan

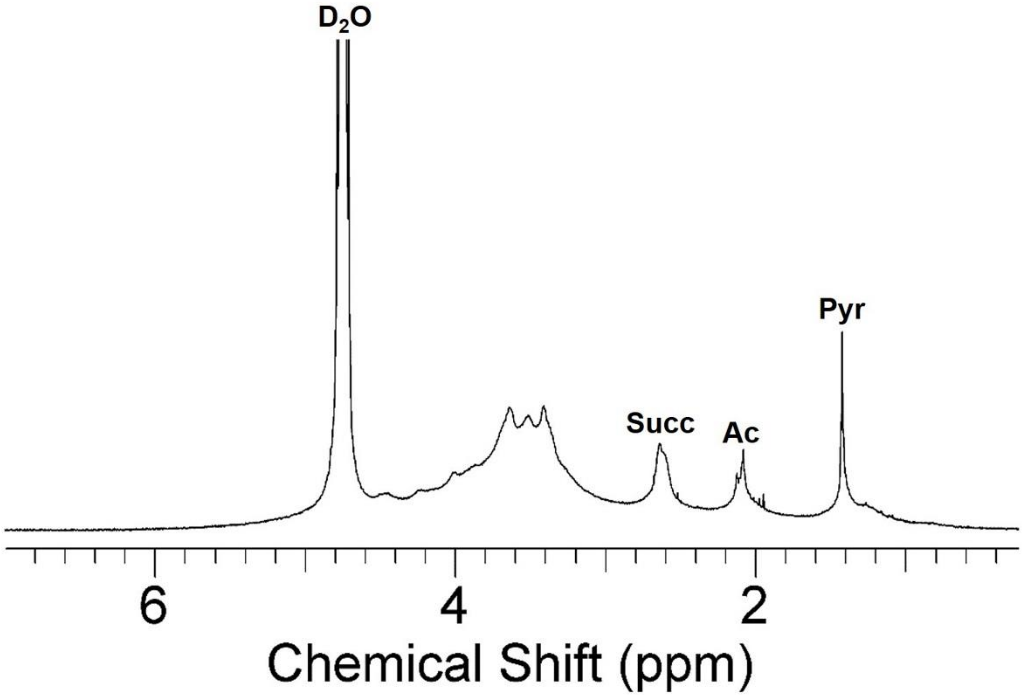

Figure S2. 500 MHz ^{1}H NMR spectra of the succinoglycan isolated from *S. meliloti* Rm 1021. The chemical shift peak at 1.43 ppm represented methyl protons of the pyruvate (Pyr);

the peaks with shifts at 2.08 ppm represented methyl protons of acetyl groups (Ac); the broad peak at 2.64 ppm represented the methylene protons of the succinate groups (Succ).

3. Photographs of gelation test

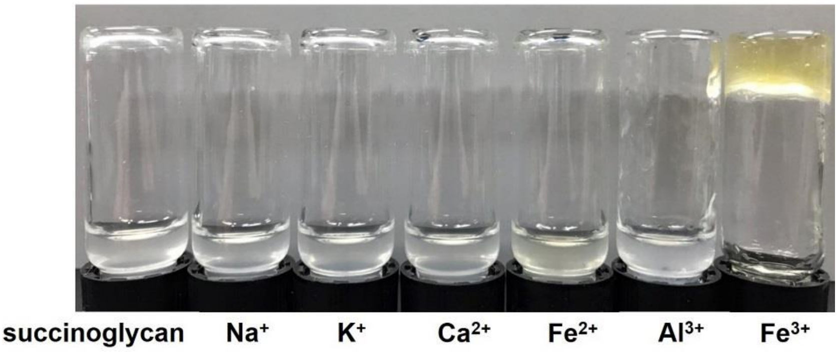

Figure S3. Photographs of gelation test of metal solution treated succinoglycan and original succinoglycan.

4. Fe-SEM analysis

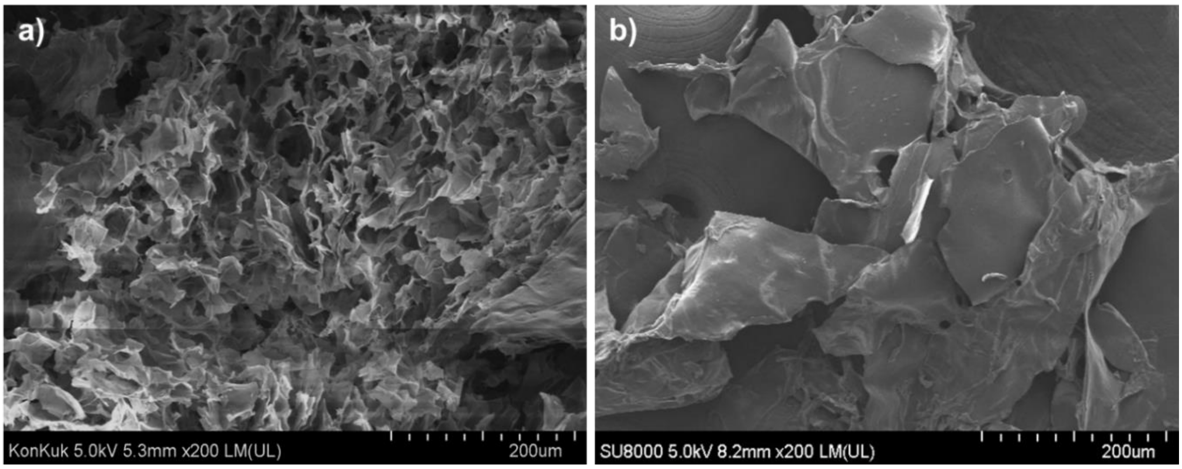

Figure S4. SEM images of Fe^{3+}-SG hydrogel beads surface view of before release (a), and after release (b) of Congo red.

5. Cytotoxicity study

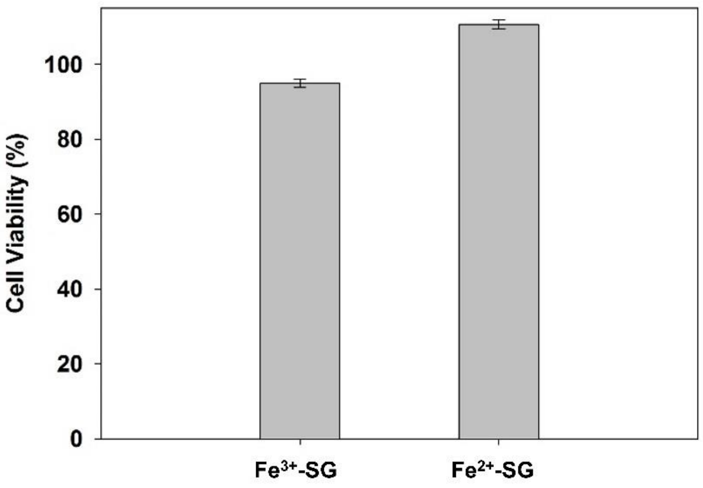

Figure S5. Cell viability of HEK 293 cells after treatment with Fe^{3+}-SG and Fe^{2+}-SG.
